# Early Postmenopausal Fragility Fractures and Elevated IgE: Two Cases Suggesting Hyper-IgE Syndrome and a Novel Adverse Reaction to Romosozumab

**DOI:** 10.1007/s00223-025-01428-z

**Published:** 2025-08-30

**Authors:** Lucy Collins, Peter R. Ebeling

**Affiliations:** 1 Department of Medicine, School of Clinical Sciences, Monash University, Melbourne, Victoria Australia; 2 Department of Endocrinology, Monash Health, Melbourne, Victoria Australia; 3 Department of Endocrinology and Diabetes, Western Health, Melbourne, Victoria Australia

**Keywords:** Secondary osteoporosis, Hyper-IgE syndrome, Anti-resorptive, Romosozumab

## Abstract

Severe, treatment-refractory or early-onset osteoporosis should prompt evaluation for secondary causes. Hyper-IgE syndrome (HIES) is a rare primary immunodeficiency disorder characterised by markedly elevated serum IgE, recurrent infections and skeletal anomalies, including osteoporosis and increased fracture burden. We present two cases of severe osteoporosis in early postmenopausal women. Both women exhibited markedly elevated IgE levels, raising the possibility of underlying HIES. Case 1, despite anabolic and anti-resorptive treatment, experienced multiple fragility fractures, with fracture burden out of keeping with bone mineral density. Case 2 did not respond to bisphosphonate therapy and developed a severe erythematous skin reaction following romosozumab therapy. Both cases highlight the importance of evaluating for secondary causes of osteoporosis. The novel reaction to romosozumab in Case 2 raises questions about its use in patients with immune dysregulation.

## Introduction

Osteoporosis is a common skeletal disorder characterised by compromised bone strength due to deterioration of bone microarchitecture and decreased bone mineral density, resulting in an increased susceptibility to fragility fractures. Although postmenopausal osteoporosis is most common, evaluation for secondary causes is important, particularly in men and in patients presenting with early-onset, severe, treatment-refractory disease or without typical risk factors [1]. Secondary causes include endocrine, inflammatory, gastrointestinal, renal and genetic conditions, amongst others. We describe two cases of severe osteoporosis in early postmenopausal women, both with markedly elevated IgE levels, raising the possibility of underlying Hyper-IgE syndrome (HIES). Case 2 developed a severe erythematous skin reaction following romosozumab treatment, a previously unreported finding.

## Case Report

### Case 1

A 56-year-old woman was investigated and treated for severe osteoporosis. Her medical history included asthma, dysfunctional breathing, lip melanoma, hypothyroidism and hyperlipidaemia. Her regular medications were thyroxine, rosuvastatin, topical estradiol/norethisterone acetate patch and inhaled fluticasone furoate/vilanterol. She did not have a family history of osteoporosis, a personal history of recurrent prior infections, and reached menopause at the age of 52. She was a smoker (3–4 cigarettes/day) and consumed minimal alcohol. Her height was 169 cm, weight 67 kg, and BMI 23.5 kg/m^2^. In 2016, she sustained multiple vertebral fractures (T12, L1, L3 and L5) which required vertebroplasty. Denosumab 60 mg subcutaneously 6 months was commenced thereafter in 2016. Baseline bone mineral density (BMD) revealed osteopaenia: left femoral neck (LFN) T score (TS) − 1.0, LFN Z score (ZS) − 0.6, lumbar spine (L1-L4, LS) TS − 1.8 and LS ZS − 1.8 (Medix DR). A screen for secondary causes of osteoporosis was unremarkable, with the exception of mildly elevated PTH ((Corrected Calcium (calculated as total calcium (mmol/L) + [(40- albumin (g/L)) × 0.02] was 2.36 mmol/L (laboratory reference range 2.2–2.6), PTH 9.6 pmol/L (1.6–6.9, Siemens Atellica immunoassay), Phosphate 0.92 mmol/L (0.8–1.5), Creatinine 64 μmol/L (45–85), 25(OH)D 108 nmol/L (> 50, DiaSorin Liaison XL immunoassay), morning cortisol 241 nmol/L (172–497), tryptase 9 ug/L (< 13.5), eosinophils 0.0 × 10^9/L (0–0.5) and TSH 1.48 mU/L (0.4–4.8). Coeliac serology was negative, and serum and urine monoclonal proteins were absent. Following two years of denosumab treatment, bone turnover markers were suppressed [C-terminal telopeptide of type 1 collagen (CTx) 92 ng/L (< 800, Roche Cobas immunoassay) and procollagen type 1 amino-terminal peptide (P1NP) 9 μg/L (15–90, Roche Cobas immunoassay). Despite suppressed bone turnover markers [CTx 100 ng/L (< 800) and P1NP 14 μg/L (15–90)] and strict adherence to the 6-monthly denosumab dosing schedule, she sustained a left sacral ala fracture in 2020. Teriparatide 20 microg subcutaneously daily was added to denosumab treatment in September 2020. Despite combination teriparatide-denosumab therapy, she continued to fracture; a right scaphoid fracture in 2020 and a left pubic bone fracture in 2021. Following 18 months of combination therapy, denosumab alone was continued. An improvement in bone mineral density was noted (LFN TS -1.1, LFN ZS -0.1, distal radius (DR) TS + 1.0 and DR ZS + 2.2). In 2024, an elevated serum IgE level of 1225 kU/L (< 100) was first noted. Full blood count was normal, and serum-specific IgE *Aspergillus fumigatus* (0.06 kU/L) and *Aspergillus fumigatus* IgG (39 mg/L) were negative. In the context of severe osteoporosis, an elevated serum IgE concentration may signal an underlying aetiology contributing to bone fragility.

### Case 2

A 56-year-old woman was investigated and treated for severe osteoporosis. Her medical history included asthma, eczema and uterine fibroids. Her regular medications were inhaled tiotropium and dupilumab. She had a family history of osteoporosis and reached menopause at the age of 51. She was a lifelong non-smoker and did not consume alcohol. Her height was 156 cm, weight 51 kg and BMI 21 kg/m^2^. In 2021, she sustained seventh and eighth rib fractures following mineral trauma. Baseline bone mineral density (BMD) revealed severe osteoporosis: LS TS -3.5, LS ZS -2.5, LFN TS-2.4 and LFN ZS -1.4 (Hologic Horizon). Alendronate 70 mg weekly was commenced in October 2021.

**Fig. 1:**
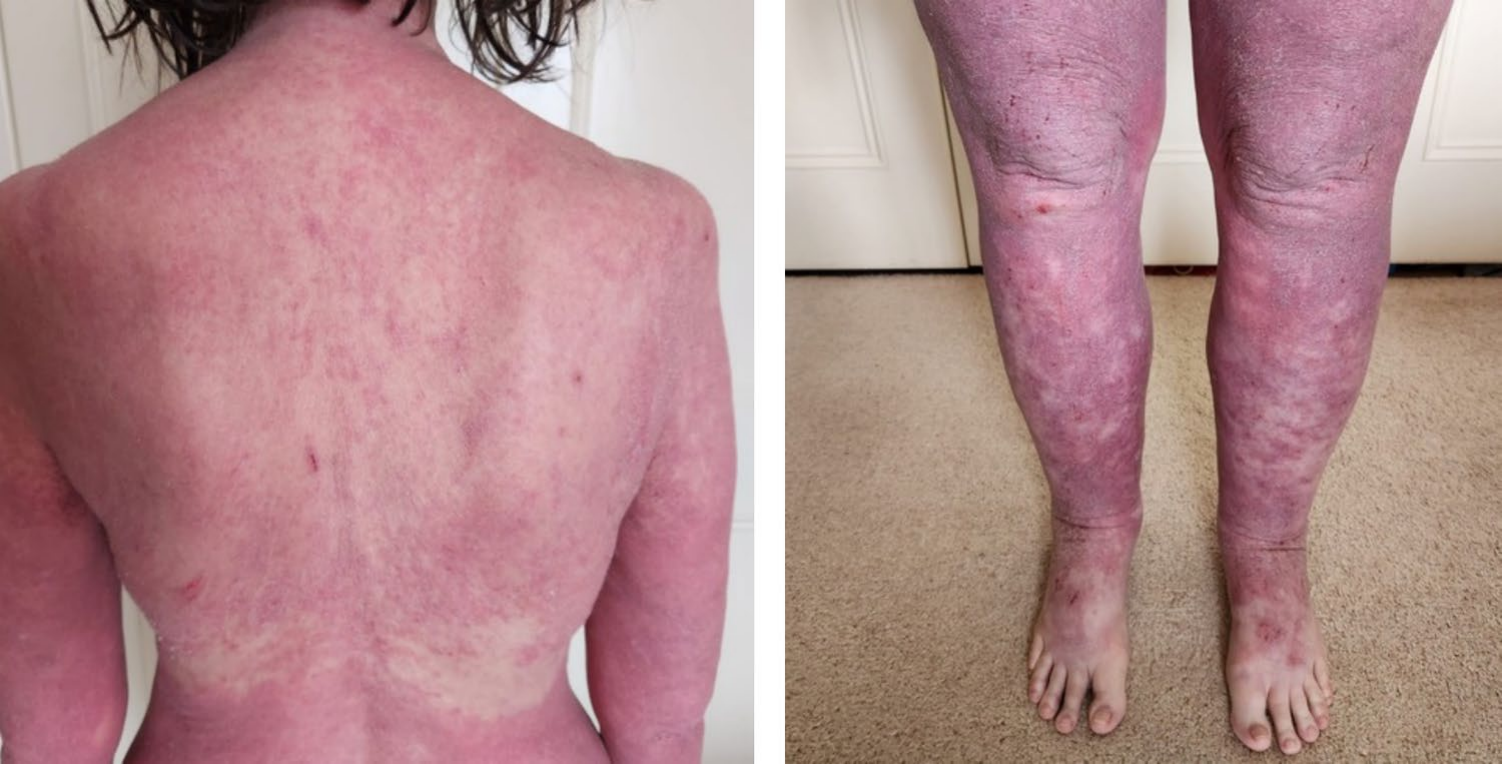
Clinical photographs demonstrating the severe widespread erythematous skin reaction following romosozumab (Case 2)

A screen for secondary causes of osteoporosis was unremarkable ((Corrected Calcium (calculated as total calcium (mmol/L) + [(40- albumin (g/L)) × 0.02] was 2.49 mmol/L (laboratory reference range 2.1–2.6), Phosphate 1.07 mmol/L (0.8–1.5), Creatinine 47 μmol/L (45–90), 25(OH)D 65 nmol/L (> 50, DiaSorin Liaison XL immunoassay), eosinophils 0.4 × 10^9/L (< 0.7) and TSH 1.18 mU/L (0.4–4.0). Coeliac serology was negative, and serum and urine monoclonal proteins were absent. Despite 12 months of alendronate, her bone mineral density continued to demonstrate severe osteoporosis (LS TS -3.6 [-0.3% since 2021 study], LS ZS -2.5, LFN TS -2.6 [-2.7% since 2021 study] and LFN ZS -1.5). In the light of this, bone turnover markers were reviewed. CTx was appropriately suppressed (130 ng/L [< 800]) indicating compliance and absorption of alendronate. Romosozumab 210 mg subcutaneously monthly was commenced and alendronate ceased. Self-resolving local injection site reactions (5-10 cm in diameter) were noted following the initial three doses of romosozumab. Following the fourth dose of romosozumab, the patient developed a severe widespread erythematous skin reaction, with associated facial swelling and fluid retention (Fig. 1). Elevated eosinophils 4.5 × 10^9/L (0–0.5) and serum IgE 21,608 kU/L (0–200) were noted. Previous IgE results were also found to be elevated in 2016: 11,704 kU/L (< 100). Histology revealed a spongiotic dermatitis with features favouring a drug eruption. She was successfully treated with prednisolone and cyclosporin for four weeks. Romosozumab was ceased, and denosumab commenced.

## Discussion

One in three women and one in five men aged 50 years or older will sustain an osteoporotic fracture [2]. Anti-resorptive medications, bisphosphonates and denosumab, have been effective treatments for osteoporosis for more than 30 years. Anabolic treatments, teriparatide and romosozumab, have demonstrated superiority in reducing fracture incidence compared with anti-resorptive agents and are recommended for patients at highest fracture risk [3, 4]. Although postmenopausal osteoporosis predominates, between 30 and 80% of men and women have a cause of secondary osteoporosis [1]. Such causes include endocrine disease, chronic inflammation, gastrointestinal conditions, glucocorticoids, chronic kidney disease, neuromuscular conditions, nutritional, malignancy and genetic conditions. The two cases highlight the importance of considering and screening for rare secondary causes of osteoporosis in patients with early-onset, treatment-refractory, or severe osteoporosis. Both patients demonstrated markedly elevated serum IgE levels, with clinical features raising the possibility of underlying Hyper-IgE syndrome (HIES). In Case 1, extensive minimal trauma fractures occurred despite treatment with both anti-resorptive and anabolic agents and appeared discordant with the patient’s bone mineral density. Although Case 1 had long-term inhaled glucocorticoid exposure for asthma, this is unlikely to fully explain the severity and treatment resistance of her osteoporosis. In Case 2, severe osteoporosis failed to respond to bisphosphonate therapy, and the patient developed a novel and severe erythematous skin reaction following romosozumab, raising questions about the safety of some anabolic therapies in patients with potential immune dysregulation.

Hyper-IgE syndrome is characterised by eczema, elevated serum IgE (> 2000 IU/L) and recurrent infections (skin/lung). Other features include skeletal anomalies, scoliosis, osteoporosis, pathological fractures and hyperextensibility [5]. Most (> 90%) cases of autosomal dominant Hyper-IgE syndrome (AD-HIES) are secondary to a loss-of-function mutation in signal transducer and activator of transcription 3 (STAT3) [6]. Autosomal recessive HIES (AR-HIES) is a distinct clinical entity, lacking the connective tissue and skeletal complications associated with AD-HIES. Mutations in the dedicator of cytokinesis-8 gene (DOCK8) have been identified in patients with AR-HIES [7]. The original clinical HIES scoring system was proposed by the National Institute of Health in 1999, based on genetic linkage studies [8]. Further scoring systems have been developed to distinguish patients with and without STAT3 mutations [9].

In individuals with HIES, the degree of fracture burden does not correlate strongly with bone mineral density [5, 10]. As demonstrated in Case 1, numerous minimal trauma fractures may occur that appear discordant with the bone mineral density. Nonetheless, rates of osteoporosis are significant in adults with HIES (~ 80%) and between 40 and 80% of adults in reported HIES cohorts have experienced fragility fractures of long bones, pelvis and ribs [10, 11]. Possible causes of low bone mineral density and increased risk of fracture include chronic inflammation and increased osteoclast function [12]. Osteoclasts generated from monocytes from HIES patients (with STAT3 mutation) exhibit higher levels of bone resorption compared with those from healthy controls [13]. In addition, STAT3-deficient mice have increased osteoclast numbers and activity resulting in osteoporosis [14]. However, the lack of correlation between fractures and bone mineral density suggests that additional contributing factors are likely involved [10]. Limited case reports have demonstrated efficacy for bisphosphonates in the treatment of minimal trauma fractures associated with AD-HIES [15].

 Romosozumab, a monoclonal antibody that inhibits sclerostin, is a potent anabolic agent that exerts the dual effects of reducing bone resorption and increasing bone formation [16]. Romosozumab has demonstrated efficacy compared with both placebo and alendronate in clinical trials examining the incidence of new fractures and changes in BMD [16, 17]. Teriparatide, another anabolic treatment option for osteoporosis, exerts its effect via the parathyroid hormone receptor-1 to stimulate coupled bone remodelling and decrease osteoblast apoptosis. Whilst teriparatide was well tolerated in Case 1, Case 2 developed a severe erythematous skin reaction following the fourth dose of romosozumab. Injection site reactions occur in approximately 5% of romosozumab-treated participants and are generally mild [16, 17]. The severity of the reaction observed in Case 2 appears disproportionate to the published safety data. Potential mechanisms include impaired Th17 cell numbers and function, increased risk of Type 1 hypersensitivity reactions (IgE mediated) and the production of anti-romosozumab antibodies that may trigger immune complex-mediated reactions. Beyond local reactions, romosozumab has been associated with cardiovascular events. Compared with alendronate, patients receiving romosozumab reported more serious cardiovascular adverse events (2.5% vs 1.9%, OR 1.31; 95% CI 0.85–2.00), cardiac ischaemic events (0.8% vs. 0.3%, OR 2.65; 95% CI 1.03–6.77) and cerebrovascular events (0.8% vs. 0.3%, OR 2.27, 95% CI 0.93–5.22) [17]. Accordingly, romosozumab is contraindicated in patients with a history of myocardial infarction or stroke [4].

This case report highlights two rare but important clinical observations: elevated serum IgE levels in two postmenopausal women with severe osteoporosis raising the possibility of underlying HIES, and an unexpected severe erythematous drug reaction to romosozumab. Ongoing clinical management includes formal genetic testing to confirm the diagnosis, as well as comprehensive evaluation of family members. Further research is needed to understand the safety profile of anabolic agents like romosozumab in patients with immune dysregulation.

## Learning Points

1. Early-onset, severe, or treatment-refractory osteoporosis should prompt consideration of secondary causes, including rare immunological or genetic disorders.
2. Markedly elevated serum IgE levels in adults with osteoporosis may suggest underlying Hyper-IgE syndrome, which can contribute to skeletal fragility independent of bone mineral density
3. Anabolic therapies, such as romosozumab, may elicit unexpected adverse reactions in patients with immune dysregulation, highlighting the need for careful monitoring and individualised treatment selection.
